# Gearbox Composite Fault Diagnosis Method Based on Minimum Entropy Deconvolution and Improved Dual-Tree Complex Wavelet Transform

**DOI:** 10.3390/e21010018

**Published:** 2018-12-26

**Authors:** Ziying Zhang, Xi Zhang, Panpan Zhang, Fengbiao Wu, Xuehui Li

**Affiliations:** 1 School of Mechanical, Electronic and Information Engineering, China University of Mining and Technology (CUMT), Xueyuan Road, Beijing 100083, China; 2 Shanxi Institute of Energy, Daxue Road, Jinzhong 030600, China

**Keywords:** improved dual-tree complex wavelet transform, minimum entropy deconvolution, gearbox composite fault, frequency mixing

## Abstract

Dual-tree complex wavelet transform has been successfully applied to the composite diagnosis of a gearbox and has achieved good results. However, it has some fatal weaknesses, so this paper proposes an improved dual-tree complex wavelet transform (IDTCWT), and combines minimum entropy deconvolution (MED) to diagnose the composite fault of a gearbox. Firstly, the number of decomposition levels and the effective sub-bands of the DTCWT are adaptively determined according to the correlation coefficient matrix. Secondly, frequency mixing is removed by notch filter. Thirdly, each of the obtained sub-bands further reduces the noise by minimum entropy deconvolution. Then, the proposed method and the existing adaptive noise reduction methods, such as empirical mode decomposition (EMD), ensemble empirical mode decomposition (EEMD), and variational mode decomposition (VMD), are used to decompose the two sets of simulation signals in comparison, and the feasibility of the proposed method has been verified. Finally, the proposed method is applied to the compound fault vibration signal of a gearbox. The results show the proposed method successfully extracts the outer ring fault at a frequency of 160 Hz, the gearbox fault with a characteristic frequency of 360 Hz and its double frequency of 720 Hz, and that there is no mode mixing. The method proposed in this paper provides a new idea for the feature extraction of a gearbox compound fault.

## 1. Introduction

In recent years, gearbox compound fault diagnosis has attracted wide attention [1,2,3]. Composite fault is one in which two or more interrelated, cross-over faults occur simultaneously on a mechanical device. Under the influence of factors such as the degree of damage at the fault location and the transmission path of the fault characteristic signal, coupled with the interference of the background noise, the vibration signal collected by the sensor has an imbalance between the fault components. The characteristics of weak faults are usually covered by strong faults or noises. In the process of transmission, high-frequency energy is weakened, and missed diagnosis or misdiagnosis is prone to occur. Especially in the case of variable speed and load, the coupling of the complex fault characteristics brings great challenges to the fault diagnosis of mechanical equipment. Therefore, compound fault diagnosis in mechanical equipment is a major difficulty in the field of mechanical fault diagnosis at present. Choosing the appropriate noise reduction algorithm and mode separation algorithm is the key factor for the accurate extraction of composite fault features.

At present, methods such as empirical mode decomposition (EMD), ensemble empirical mode decomposition (EEMD), variational mode decomposition (VMD), discrete wavelet transform (DWT), and dual-tree complex wavelet transform (DTCWT) are widely used in gearbox composite fault feature extraction, and have achieved good results.

EMD is a method to deal with non-linear and non-stationary signals [4,5,6]. The method decomposes the signal into several intrinsic mode functions adaptively, according to the characteristics of the signal itself. Each intrinsic mode function contains different frequency components of the original signal. However, EMD also has mode mixing and endpoint effects. In order to overcome the shortcomings of EMD, Huang et al. proposed EEMD [7]. It effectively solves the mode mixing problem of EMD by adding a specific Gaussian white noise sequence to the vibration signal. Therefore, it can accurately reflect the characteristic information of the original signal [8]. However, the decomposition effect of EEMD depends on the added white noise amplitude and the number of filtering times. The accuracy of the decomposition results depends, to a large extent, on experience [9]. The improper selection of white noise will lead to mode mixing. Variational mode decomposition (VMD) was proposed by Dragomiretskiy [10] et al. This method adaptively separates the frequencies of the signals from the low to the high, thus avoiding frequency mixing successfully. However, the decomposition of level K and penalty factor of VMD need to be determined in advance. The improper selection of the K value will easily lead to under-decomposition or over-decomposition, and can further lead to misdiagnosis.

Wavelet transform (WT) is a powerful tool for processing non-linear and non-stationary signals. WT performs a multi-scale time-frequency analysis of signals by stretching and translating the transform [11,12]. WT mainly refers to continuous wavelet transform (CWT) and discrete wavelet transform (DWT). CWT has a low computational efficiency, while DWT has a high computational efficiency and is widely used in fault diagnosis [13]. Kingsbury [14,15] and Selesnick [16] et al. proposed dual-tree complex wavelet transform (DTCWT) based on DWT. Not only can the excellent characteristics of discrete wavelet transform be preserved, but DTCWT also has approximate translational distortion and small frequency mixing [17]. Therefore, DTCWT is widely used in the field of fault diagnosis. Chen [18] et al. proposed a new method based on the DTCWT threshold denoising and Laplacian eigenmaps (LE) for planetary gear fault identification, which proves the good denoising effect of DTCWT. Van M [19] et al. combined DTCWT with EMD to extract the temporal features of the signal, and then used the K-nearest neighbors and genetic algorithms to accurately identify the fault features. Hu [20] et al. combines Multiscale noise tuning stochastic resonance (MSTSR) with DTCWT to diagnose the wind system faults. Sun [21] et al. used DTCWT to extract the features of multi-scale signals. In addition, the convolution neural network (CNN) method was used to automatically identify the fault features from multi-scale signal features. Xiao [22] et al. decomposed and reconstructed the characteristic signal, and the dual-tree complex wavelet energy entropy was obtained from the reconstructed coefficients to form the feature vector of the fault diagnosis, and then used a support vector machine for fault identification. Chen [23] proposed a fault diagnosis method for planetary gears based on the entropy feature fusion of dual-tree complex wavelet transform (DTCWT) and the optimized kernel Fisher discriminant analysis (OKFDA). The number of layers decomposed by DTCWT is very important for fault feature extraction. The value depends on experience selection. Inappropriate values may cause over-decomposition or under-decomposition. At present, there are few studies on the adaptive decomposition layer of DTCWT. Although DTCWT reduces the frequency mixing to some extent, the frequency mixing still inevitably appears in the sub-bands reconstructed by DTCWT. It is more common to use wavelet threshold noise reduction to reduce frequency mixing [24,25]. Shao et al. obtained the main frequency component signals of each sub-band by EMD, and successfully overcame the frequency mixing of DTCWT. However, the EMD decomposition of each sub-band will produce more invalid Intrinsic Mode Functions (IMFs).

However, the sub-band obtained by dual-tree complex wavelet decomposition still contains a lot of noise, which needs to be de-noised. Minimum entropy deconvolution is a common method of noise reduction in fault diagnosis. It can not only reduce the noise of the signal, but also enhance the impact component. ENDO [26] is the first one to use minimum entropy deconvolution to reduce the noise of vibration signals to diagnose the gear flaking and crack faults of a gearbox. Sawalhi et al. [27] used minimum entropy deconvolution to detect faults of rolling bearings. Li et al. [28] proposed a method based on time-delayed feedback monostable stochastic resonance (TFMSR) system and minimum entropy deconvolution (MED) to achieve the purpose of fault detection for rolling bearings. He et al. [29] combined minimum entropy deconvolution and spectral kurtosis to achieve the purpose of multiple fault diagnosis of rotating machinery. Li et al. [30] used minimum entropy deconvolution as the pre-filter of the sinusoidal wave synthesis (MEDSS) filter, which improved the fault detection performance of the conventional sinusoidal wave synthesis (SS) method.

Aimed at the problems that the number of layers decomposed by DTCWT requires prior to determination and frequency mixing, an improved dual-tree complex wavelet transform is proposed in this paper. Firstly, the maximum number of decomposition levels of DTCWT is initialized according to the signal length and the filter order, and adaptively determines the number of decomposition layers according to the reconstructed sub-band and the correlation coefficient matrix of the original signal. Secondly, the effective sub-bands are adaptively determined from the reconstructed sub-bands according to the dominant frequency and the correlation coefficient. After the valid sub-band is determined adaptively, each sub-band is removed in the frequency mixing by the filtering method, to ensure that each sub-band contains only a unique frequency component. Thirdly, each of the obtained sub-bands further reduces the noise by minimum entropy deconvolution. Then, by comparing the results with EMD, EEMD, and VMD, it was verified that the proposed method could not only determine the decomposition level and select the effective sub-band adaptively, but also eliminate the frequency mixing in EMD, EEMD, and VMD. Finally, the vibration test of the gearbox test bed was carried out to verify the method, which proves that the proposed method can successfully extract the composite fault.

## 2. Dual-Tree Complex Wavelet Transform

### 2.1. The Theoretical Review

**Figure 1:**
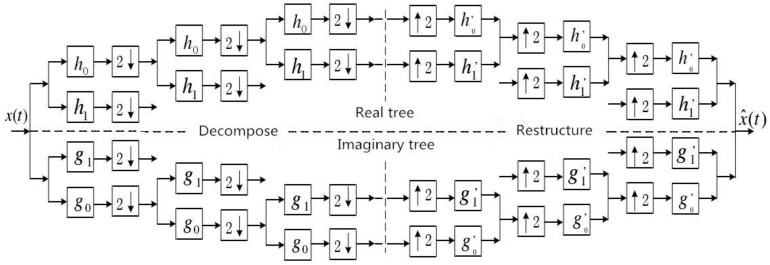
Decomposition and reconstruction process of dual-tree complex wavelet transform (DTCWT).

The dual-tree complex wavelet transform uses two parallel discrete wavelet transforms to realize the decomposition and reconstruction of the signal, which are real tree and imaginary tree, respectively. In the process of signal decomposition and reconstruction, the sampling position of the imaginary tree is always located in the middle of the real tree, so that the approximate translation invariance can be obtained and the loss of information can be reduced. Where, $h_{0}$ and $h_{1}$ are the low-pass and high-pass decomposition filters of real tree, respectively; $g_{0}$ and $g_{1}$ are the low-pass and high-pass decomposition filters of imaginary tree, respectively; $h_{0}^{\prime }$ and $h_{1}^{\prime }$ are the reconstruction filters of low-pass and high-pass real tree, respectively; and $g_{0}^{\prime }$ and $g_{1}^{\prime }$ are the low-pass and high-pass reconstruction filters of imaginary tree, respectively. The decomposition and reconstruction process of DTCWT are shown in Figure 1. In this paper, the first filter used is a 14-order Q-shift filter.

The two discrete wavelets are $\psi_{h}(t)$ and $\psi_{g}(t)$, respectively, and transforming them into the complex domain to obtain the form of the double-tree complex wavelet complex wavelet is as follows:

(1) $$\psi (t)=\psi_{h}(t)+i\psi_{g}(t)$$

where, $\psi_{h}(t)$ is the real tree wavelet, $\psi_{g}(t)$ is the imaginary tree wavelet (both are real wavelets), and *i* is a complex unit.

For real tree, the real wavelet coefficients and scale coefficients can be calculated from the inner product operation according to the wavelet transform theory, as follows:

(2) dIjRe(k)=2j2∫−∞+∞x(t)ψh(2jt−k)dt j=1,2,⋯,J

(3) cIJRe(k)=2J2∫−∞+∞x(t)ψh(2jt−k)dt

where, *i* is the scale factor and *j* is the largest scale.

Similarly, for imaginary tree, its wavelet coefficients and scale coefficients are as follows:

(4) dIjIm(k)=2j2∫−∞+∞x(t)ψg(2jt−k)dt j=1,2,⋯,J

(5) cIJIm(k)=2J2∫−∞+∞x(t)ψg(2jt−k)dt

From the above formula, the wavelet coefficients and scale coefficients of DTCWT can be obtained as follows:

(6) djφ(k)=dIjRe(k)+idIjIm(k) j=1,2,⋯,J

(7) cJφ(k)=cIJRe(k)+cIJIm(k)

The wavelet coefficients and scale coefficients after signal reconstruction are as follows:

(8) cJ(t)=2J−12[∑k=−∞+∞cIJRe(k)ψh′(2Jt−k)+∑k=−∞+∞dIJIm(k)ψg′(2Jt−k)]

Wavelet coefficients and scale coefficients can be reconstructed to obtain the components of the different frequency bands. The reason that the double-tree complex wavelet has many excellent characteristics, such as translation invariance and suppression of frequency mixing, is because $\psi_{h}(t)$ and $\psi_{g}(t)$ are a Hilbert transform pair, and the two low-pass filter coefficients satisfy the sampling delay condition, as follows: In the first level decomposition, the delay between the real part and the imaginary part filter banks happens to be an interval of the sampling values, and a complementary relation is formed in the second sampling, that is, the data sampled by the imaginary part is exactly the real part not sampled.

The reconstructed signal obtained by DTCWT is as follows:

(9) $$\hat{x}(t)=\sum_{j=1}^{J}d_{j}(t)+c_{J}(t)\;\;j=1,2,\cdots ,J$$

The small translation of the input signal can lead to a great change of the wavelet coefficients on each scale of the discrete wavelet transform, that is, the discrete wavelet is sensitive to translation. Dual-tree complex wavelet transform uses two discrete wavelet transforms to construct filter banks with a sampling point delay, which not only speeds up the sampling speed, but also overcomes the translation sensitivity problem caused by the strict sampling of discrete wavelet transform.

### 2.2. Defects of DTCWT

(1) The number of decomposition layers needs to be predetermined. If the frequency components in the original signal are known, the number of decomposition layers can be determined based on the number of frequency components. If the frequency components in the original signal are unknown, the number of decomposition layers needs to be determined empirically. If there are too many decomposition layers, the low frequency sub-band will contain less information on the original signal, and more invalid sub-bands will be generated. If the number of decomposition levels is too small, the frequency components of the original signal are not effectively decomposed into different sub-bands, resulting in frequency mixing.
(2) There is frequency mixing in each sub-band. In the decomposition process of DTCWT, the sampling frequency is halved by down-sampling (selecting values at intervals), which leads to frequency mixing. Up-sampling (setting zero at intervals) during the reconstruction process also causes frequency components that do not exist in the original signal to appear in each sub-band obtained by the reconstruction. The frequency response of the first filter used in the dual-tree complex wavelet transform at a negative frequency is another reason for frequency mixing.

The simulation signal is constructed as follows:

x=sin(60πt)+sin(150πt)+sin(200πt)

**Figure 2:**
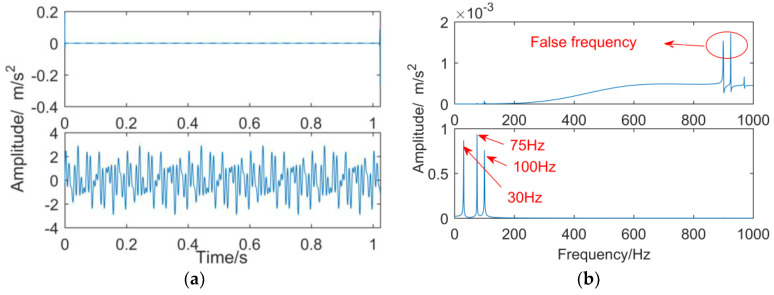
The results of DTCWT, the number of decomposition layer is one. (**a**) Time domain diagram; (**b**) frequency domain diagram.

**Figure 3:**
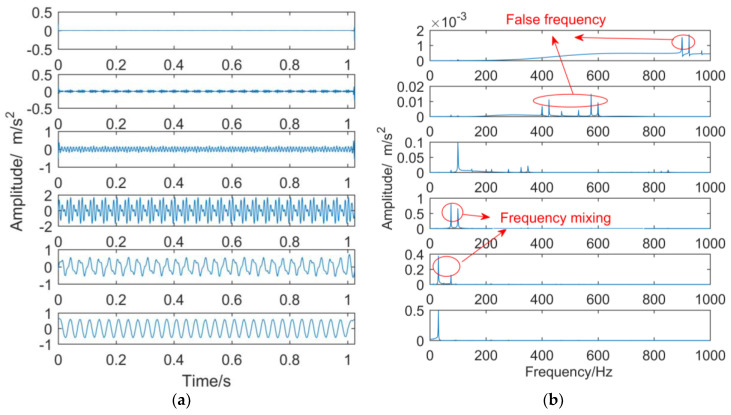
The results of DTCWT, the number of decomposition layer is six. (**a**) Time domain diagram; (**b**) frequency domain diagram.

The sampling points are 2048 and the sampling frequency is 2000 Hz. The signal is decomposed into one and six layers by DTCWT, and the results are shown in Figure 2 and Figure 3, respectively. As can be seen from Figure 2, as the number of decomposition layers is too small, frequency mixing occurs in the second layer, and a spurious frequency appears in the first layer. As can be seen from Figure 3, because of the excessive number of decomposition layers, the sixth layer is an invalid sub-band, the first layer and the second layer have false frequencies due to up-sampling, and the fourth layer and the fifth layer have frequency mixing.

## 3. Improved Dual-Tree Complex Wavelet Transform

**Figure 4:**
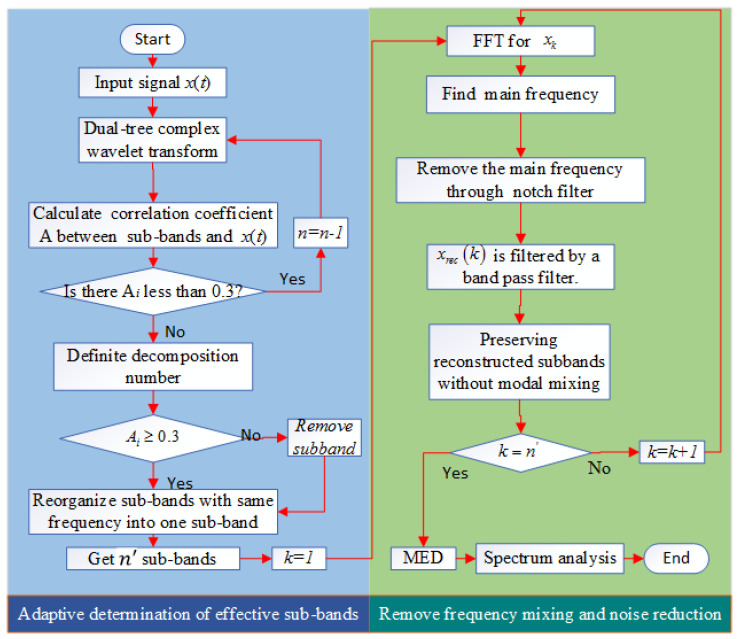
The flow chart of improved DTCWT (IDTCWT).

The improved dual-tree complex wavelet transform (IDTCWT) proposed in this paper solves the defect of DTCWT, an mainly includes the following: Firstly, not only the decomposition layers of dual-tree complex wavelet transform can be determined adaptively, but also, the effective sub-bands can be selected adaptively. Secondly, each of the obtained sub-bands is implemented to remove the frequency mixing. The flow chart is shown in Figure 4.

### 3.1. Adaptive Determination of Effective Sub-Bands

First, the decomposition layer of IDTCWT is limited by signal length and filter length. The number of decomposition layers satisfies Equation (10), as follows:

(10) $$\left\{ \begin{array}{l} n\leq \log_{2}(N/l)+1\\ \frac{N}{2^{n}}\in Z+ \end{array}\right.\;\;n\in Z+$$

where: *N* is the length of the original signal x(t) and *l* is the filter length of DTCWT. In this paper, the Q-shift filter is used in DTCWT, and it has a length of L = 14.

The steps to adaptively determine the effective sub-band of DTCWT are as follows:

I. Adaptively determine the number of decomposition layers of DTCWT

(1) Initialize the number of decomposition layers (the largest positive integer satisfying the above conditions) and record it as $n=\log_{2}(N/l)+1$.
(2) Perform DTCWT (the number of decomposition layers is N) on the original signal X, and obtain *N* + 1 sub-bands with different frequency components after reconstruction, $x_{1}(t),x_{2}(t),\cdots ,x_{n+1}(t)$; each sub-band length is *N*, which is consistent with the original signal length.
(3) DTCWT is not a complete binary tree structure, and each layer decomposition only subdivides the low-frequency part. So, the more decomposition layers, the less the low-frequency sub-band contains the original signal information, and the smaller the correlation coefficient with the original signal. Therefore, the correlation coefficient $A_{i}$ (i=1,2,⋯,n+1) between each reconstructed sub-band $x_{i}(t)$ and the original signal x(t) can be calculated. The formula of the correlation coefficient T is as follows:
  (11) $$A_{i}=\frac{\mathrm{Cov}(x_{i}(t),x(t))}{\sqrt{D(x_{i}(t))}\sqrt{D(x(t))}}=\frac{E(x_{i}(t)-\mu_{x_{i}(t)})(x(t)-\mu_{x(t)})}{\sqrt{D(x_{i}(t))}\sqrt{D(x(t))}}$$
  where, $\mathrm{Cov}(x_{i}(t),x(t))$ is the covariance of $x_{i}(t)$ and x(t), E(*) is the mathematical expectation, μ is the sample mean, and $D(x_{i}(t))$ and D(x(t)) are the squared difference between $x_{i}(t)$ and x(t). The correlation coefficient matrix ${A=\{A}_{1},A_{2},\cdots ,A_{n+1}\}$, the relationship between the correlation coefficient size, and correlation is shown in Table 1.
  From Table 1, when the absolute value of the correlation coefficient is between 0.3 and 1, it indicates that the correlation between the two data sequences is strong. When $A_{i}\geq 0.3$, it indicates that the sub-band $x_{i}(t)$ contains more information in the original signal and is a valid sub-band. When $A_{i}<0.3$, it indicates that the sub-band $x_{i}(t)$ contains less information in the original signal and is an invalid sub-band.
(4) Judging whether *n* = $\log_{2}(N/l)+1$, if so, Step (5) is executed, if not, Step (5) is skipped, and the effective sub-band is determined adaptively.
(5) When the number of decomposition layers is large enough, the correlation coefficient of the sub-band must be less than 0.3, so the number of decomposition layers is adaptively determined by the correlation coefficients of the adjacent two layers. Let j=n,n−1,⋯,1, when $A_{j}$ and $A_{j+1}$ satisfy the following formula:
  (12) $$(A_{j}-0.3)(A_{j+1}-0.3)\leq 0$$

Update the decomposition layer *n* to n=j−1, and repeat Steps (1)–(4). So far, the adaptive determination of the decomposition layers of DWCWT is completed.

Although the number of decomposition layers has been adaptively determined, the above constraints cannot fully guarantee that the correlation coefficients of each sub-band of the adaptive decomposition satisfy $A_{i}\geq 0.3$, and the same frequency components may appear in different sub-bands after reconstruction. Therefore, we need to screen the obtained sub-bands and determine the effective sub-bands adaptively.

II. Adaptively determine the effective sub-bands of DTCWT

(1) Calculate the correlation coefficient between the *n* + 1 sub-bands adaptively obtained by DTCWT and the original signal. Removing the sub-bands with a correlation coefficient less than 0.3, and obtaining the $n^{\prime }$ sub-bands $x_{1}(t),x_{2}(t),\cdots ,x_{n}^{\prime }(t)$, where $n^{\prime }<n$.
(2) Each sub-band performs Fourier transform, and the frequency corresponding to the maximum amplitude in the amplitude spectrum is recorded as the main frequency $f_{1},f_{2},\cdots ,f_{n\prime }$.
(3) Find the sub-bands with the same dominant frequency and reorganize into one sub-band. Finally, $n^{\prime \prime }$ sub-bands are obtained, which are called the effective sub-bands of DWCWT.

In this way, the adaptive determination of the effective sub-band is achieved. However, frequency mixing will inevitably occur in the reconstructed sub-bands because of the down-sampling and up-sampling in the process of decomposition and reconstruction of DWCWT, so it is necessary to remove the frequency mixing in the reconstructed sub-bands.

### 3.2. Remove the Frequency Mixing of Each Sub-Band

**Figure 5:**
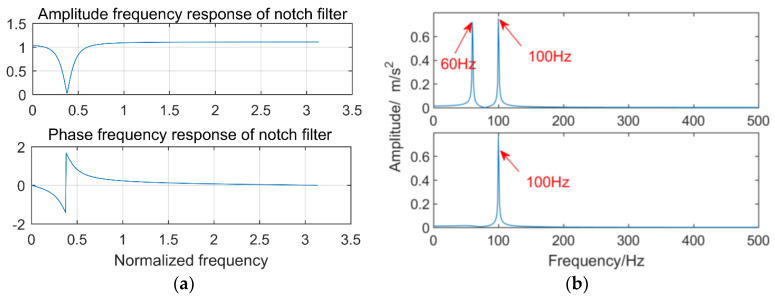
Notch filter: (**a**) frequency response of the notch filter; (**b**) effect of notch filter.

Frequency mixing inevitably occurs in the sub-bands reconstructed by DWCWT, so the remove frequency mixing (RFM) operation is performed on the effective sub-bands. The steps are as follows:

(1) Let k=1 and perform fast Fourier transform on $x_{k}(t)$.
(2) The frequency corresponding to the largest amplitude in the amplitude spectrum is the main frequency component of $x_{k}(t)$, which is recorded as $f_{\max}$.
(3) Remove the main frequency by the trap filter. The system function of the notch filter is as follows:
  (13) $$H(z)=\frac{1-(2\cos\omega_{0})z^{-1}+z^{-2}}{1-(2r\cos\omega_{0})z^{-1}+r^{2}z^{-2}}$$
  where $\omega_{0}=\frac{2\pi f_{0}}{fs}$ is the notch digital frequency; $f_{0}$ is the notch frequency, that is the main frequency component; fs is the sampling frequency; and r is a constant. Here, the sampling frequency is 1000 Hz, the notch frequency is 60 Hz, and the constant r is 0.9. The amplitude–frequency response and phase–frequency response of the notch filter are shown in Figure 5a. We construct a signal superimposed by two sinusoidal signals with frequencies of 100 Hz and 60 Hz. The notch frequency is 60 Hz and the sampling points are 1024. The other parameters are consistent with the front. The FFT results before and after filtering are shown in Figure 5b. It can be observed that the frequency components of 60 Hz are filtered out.
  After filtering the main frequency notch filter, the signal ${\hat{x}}_{k}(t)$ is obtained.
(4) Get a reconstruction signal $x_{rec}(k)=x_{k}(t)-{\hat{x}}_{k}(t)$.
(5) Calculate the correlation coefficient $A_{k}$ between $x_{rec}(k)$ and $x_{k}(t)$. If $A_{k}\geq 0.3$, let $x_{k}(t)={\hat{x}}_{k}(t)$ and store $x_{rec}(k)$ in the two-dimensional matrix $X_{rec}(k)(m)$, until the correlation coefficient $A_{k}<0.3$. The frequency components in $x_{i}(t)$ are extracted.
(6) In order to ensure that the frequency components other than the main frequency are filtered out, $x_{rec}(k)$ is filtered by a band pass filter.
(7) Judge whether k is equal to $n^{\prime \prime }+1$, if not, let k=k+1, repeat Steps (1)–(6) until $k=n^{\prime \prime }$.

### 3.3. Noise Reduction for Each Sub-Band

The sub-bands obtained by the decomposition of the double-tree complex wavelet still contain a certain degree of noise. In order to achieve the optimal decomposition result, the sub-bands need to be further denoised. Minimum entropy deconvolution is a commonly used signal denoising method in the field of fault diagnosis. The core is to find an inverse filter that minimizes the noise component while enhancing the impact component.

The flow of minimum entropy deconvolution algorithm is as follows:

- Step 1: Calculate the Toeplitz autocorrelation matrix R and initialize the optimal FIR filter coefficients $g^{\left(0\right)}$.
- Step 2: According to Equation (14), the output signal $y^{\left(k\right)}$ is calculated by using input signal x(n) and the optimal FIR filter coefficients $g^{\left(k\right)}$, where k is the *k*th iteration.
  (14) $$y\left(n\right)=\sum_{l=1}^{L}g\left(l\right)x\left(n-l\right)$$
  where L is the length of the optimal FIR filter.
- Step 3: Calculate the left side of Equation (15) $b^{\left(k+1\right)}$ and solve the new optimal FIR filter coefficients $g^{\left(k+1\right)}=R^{-1}b^{\left(k+1\right)}$.
  (15) b=gR
  where *b* represents cross-relationship of the input and output signal of the FIR filter; R is a Toeplitz autocorrelation matrix of the input signal, and g is the column vector of the required FIR filter coefficients.
- Step 4: Calculate the error criterion, $E\left(err\right)={\left\|g^{\left(k+1\right)}-g^{\left(k\right)}\right\|}^{2}$
- Step 5: If the E(err) > tolerance, then go to Step 2 with a new FIR filter coefficient of $g^{\left(k+1\right)}$ for the next iteration, from Step 2 to 4; otherwise, stop the process.

## 4. Simulation Signal Analysis

**Figure 6:**
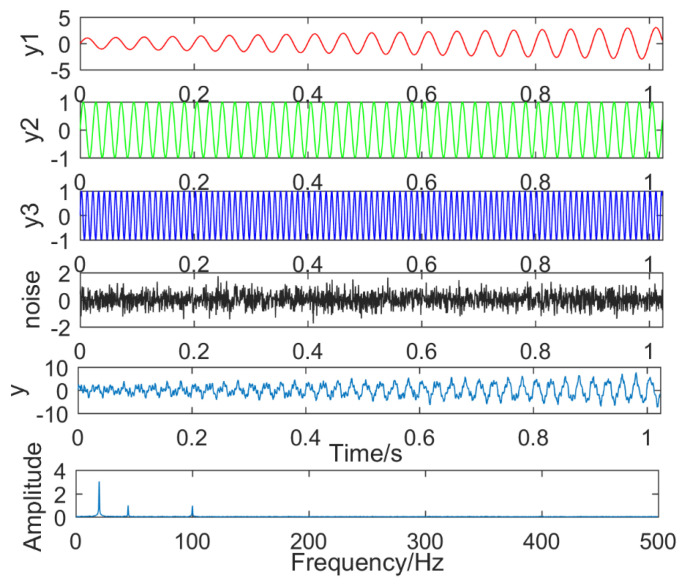
Simulation signal.

To verify the feasibility of the proposed method, the following simulation signals are constructed. The number of sampling points is 2048, and the sampling frequency is 2000 Hz.

(16) $$\begin{array}{l} y_{1}(t)=(1+2t)\sin(40\pi t)\\ y_{2}(t)=\sin(90\pi t)\\ y_{3}(t)=\sin(200\pi t) \end{array}$$

where, noise(t) is the Gaussian white noise, and y(t) is a simulation signal, which includes $y_{1}(t)$, $y_{2}(t)$, $y_{3}(t)$, and noise. The frequencies are 20 Hz, 45 Hz, and 100 Hz, respectively, and their time domain waveforms and the result of y(t) executing FFT is shown in Figure 6. The frequency component of $y_{1}(t)$ is 20 Hz and has a tendency, the frequency component of $y_{2}(t)$ is 45 Hz, and the frequency component of $y_{3}(t)$ is 100 Hz.

(1) Results of EMD

**Figure 7:**
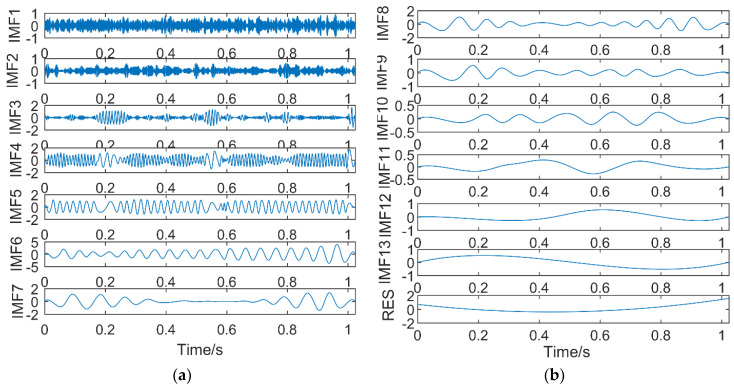
The decomposition results of empirical mode decomposition (EMD): (**a**) IMF1–IMF7; (**b**) IMF8–RES.

The simulation signal is decomposed by EMD, and it is decomposed into 14 sub-bands, including 13 intrinsic mode functions and a residual. The decomposition results are shown in Figure 7. Figure 7a shows IMF1–IMF7, and Figure 7b shows IMF8–Residual (RES).

It can be seen from Figure 7a that IMF1 and IMF2 are high frequency noise components, IMF3 and IMF4 belong to the same time scale and are in mixing state, IMF5 also appears mixing phenomenon, and IMF6 is close to the *y*_1_(*t*) component. Therefore, except for IMF6, other IMFs all have frequency mixing. Figure 7b shows that the latter six IMFs and the residual do not contain the frequency components in the simulation signal *y*(*t*). In conclusion, the decomposition of the simulated signal by EMD not only produces more eigenmode functions, but also, the frequency mixing is more serious.

(2) Results of EEMD

**Figure 8:**
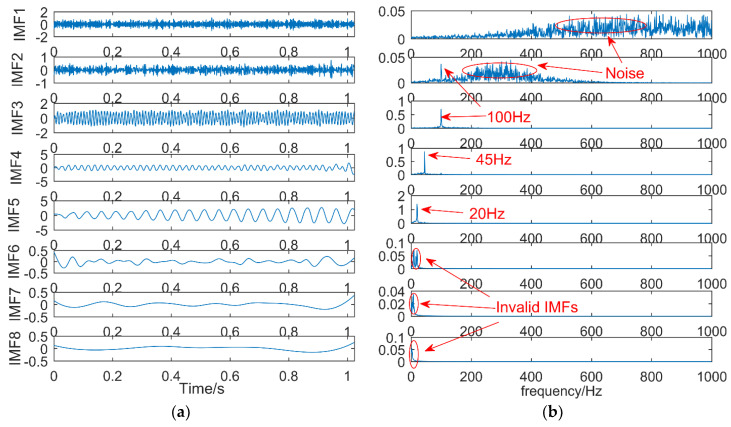
The decomposition results of ensemble empirical mode decomposition (EEMD): (**a**) time domain; (**b**) frequency domain.

The simulation signal is decomposed into eleven IMFs and one RES by EEMD, and the first eight IMFs are selected. The result is shown in Figure 8. IMF1 is the noise and IMF2 also contains a lot of noise. IMF3–IMF5 contains the frequency components in the simulation signal, the frequencies are 100, 45, and 20 Hz respectively, and IMF6–IMF8 are the invalid IMFs. Compared with EMD, EEMD can better decompose the frequency components in the simulation signal in turn, and the mode mixing is small. However, EEMD has also produced more invalid IMFs.

(3) Results of VMD

**Figure 9:**
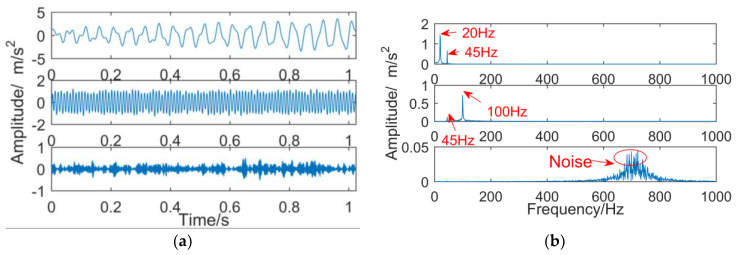
The decomposition results of variational mode decomposition (VMD): (**a**) time domain; (**b**) frequency domain.

When the number of decomposition layers is three and the penalty factor is 2000, the simulation signal is decomposed by VMD, and the result is shown in Figure 9. As shown in Figure 9, the time-domain waveform corresponding to the frequency of 20 Hz in the simulation signal *y*(*t*) has been seriously distorted, and the frequency of 45 Hz appears in the first and second layers simultaneously. There are 20 and 45 Hz in the spectrum of the first sub-band, so there is serious frequency mixing, and the third sub-band is noise. Compared with the decomposition results of EEMD, VMD fails to separate the different time scales in the simulation signal, and the frequency mixing is serious.

(4) Results of IDTCWT

**Figure 10:**
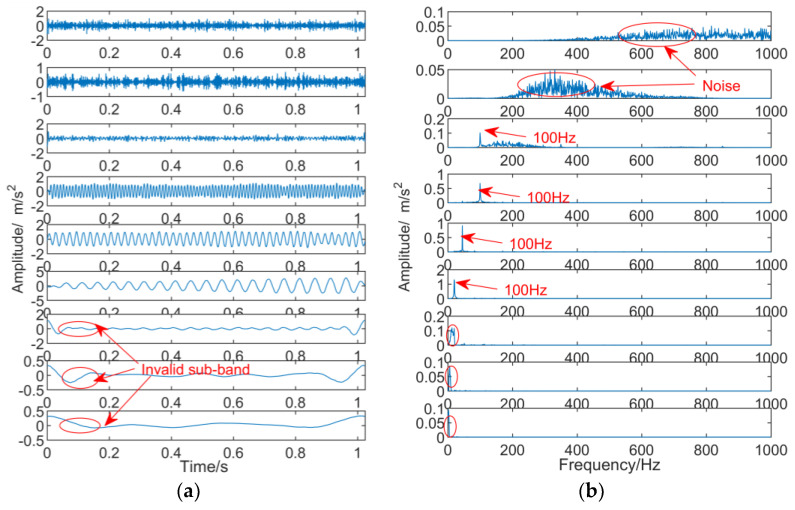
The decomposition results of dual-tree complex wavelet transform: (**a**) time domain; (**b**) frequency domain.

When the maximum number of decomposition layers is eight, the simulated signal is decomposed by the improved dual-tree complex wavelet transform, and the obtained nine sub-bands and their frequency spectrum are shown in Figure 10. Sub-band one and two are noises, and the correlation coefficients with the simulated signals are 0.2054 and 0.1728, respectively, which should be removed. Sub-bands four, five, and six contain frequency components in the simulation signal, and the latter two sub-bands are invalid sub-bands. Therefore, the number of decomposition layers needs to be determined adaptively.

**Figure 11:**
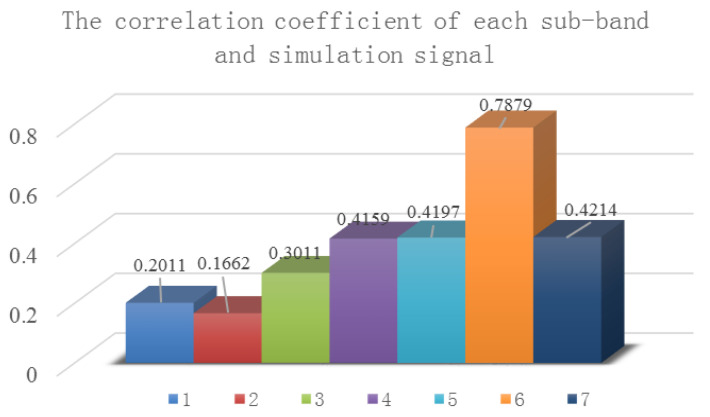
The correlation coefficient of each sub-band and simulation signal.

According to Equation (12), the decomposition level is six layers adaptively, and seven sub-bands are obtained. The correlation coefficients between the sub-bands and simulation signals are shown in Figure 11. The main frequency of each sub-band is shown in Table 2. The main frequencies of sub-bands three and four are 100 Hz, and the main frequencies of sub-bands six and seven are 20 Hz. The correlation coefficient between sub-band one and two with a simulation signal is less than 0.3, which is a noise component and can be eliminated directly. The final self-adaptively selected sub-bands are the recombined sub-band after recombination of sub-bands three and four, sub-band five, and the recombined sub-band after the recombination of sub-bands six and seven.

**Figure 12:**
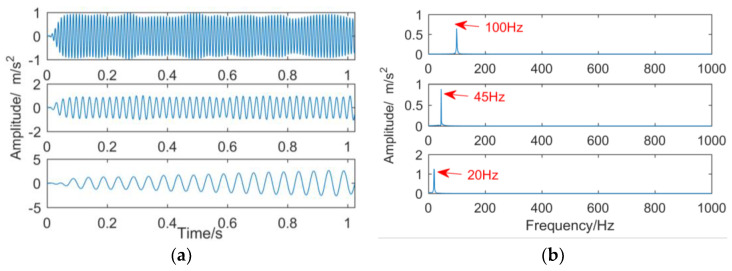
The reconstructed sub-bands of the improved dual-tree complex wavelet transform after reducing noise by minimum entropy deconvolution: (**a**) time domain; (**b**) frequency domain.

Figure 12 shows that the results of removing the frequency mixing for the selected effective sub-bands and reducing the noise in the sub-bands by minimum entropy deconvolution. The frequencies of 20, 45, and 100 Hz in the simulation signal are successfully decomposed and the noise is removed. However, there is a weak endpoint effect, which is due to the waveforms filtered by the selected filter, which is not completely consistent with the waveforms of each frequency component in the original signal. But compared with EEMD, the improved dual-tree complex wavelet transform not only adaptively determines the decomposition level, but also adaptively determines the effective sub-band. From the frequency domain, the improved dual-tree complex minor para-complex wavelet transform can adaptively separate the time scales of the original signal in turn, and there is no frequency mixing phenomenon.

**Figure 13:**
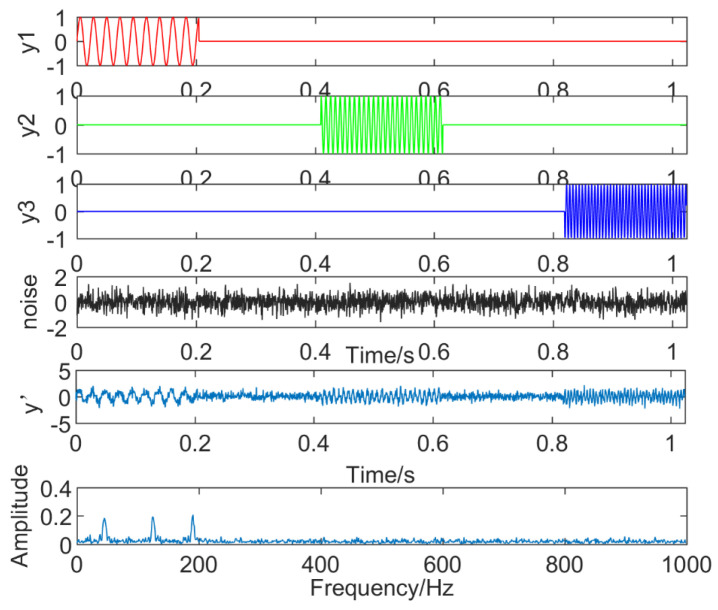
Intermittent simulation signal.

To further verify the feasibility of the improved dual-tree complex wavelet transform for decomposing intermittent signals, an intermittent simulation signal is constructed. The sampling point N is 2048 and the sampling frequency Fs is 2000 Hz.

(17) $$y(t)=y_{1}^{\prime }(t)+y_{2}^{\prime }(t)+y_{3}^{\prime }(t)+0.5*\mathrm{noise}(t)$$

(18) $$\begin{array}{l} y_{1}^{\prime }(t)=\left\{ \begin{array}{ll} \sin(90\pi t) & t\in [0,\frac{N}{5fs}]\\ 0 & t\in (\frac{N}{5fs},\frac{N}{fs}] \end{array}\right.\\ y_{2}^{\prime }(t)=\left\{ \begin{array}{ll} \sin(250\pi t) & t\in (\frac{2N}{5fs},\frac{3N}{5fs}]\\ 0 & t\in [0,\frac{2N}{5fs}]\cup (\frac{3N}{5fs},\frac{N}{fs}] \end{array}\right.\\ y_{3}^{\prime }(t)=\left\{ \begin{array}{ll} \sin(380\pi t) & t\in (\frac{4N}{5fs},\frac{N}{fs}]\\ 0 & t\in [0,\frac{4N}{5fs}] \end{array}\right. \end{array}$$

where, noise is Gauss white noise, and the time domain waveform and frequency domain graph of the simulation signal are shown in Figure 13. The frequency component of $y_{1}^{\prime }(t)$ is 45 Hz, the frequency component of $y_{2}^{\prime }(t)$ is 125 Hz, and the frequency component of $y_{3}^{\prime }(t)$ is 190 Hz.

In order to verify the feasibility of improving the dual-tree complex wavelet transform, the sub-bands obtained by the improved dual-tree complex wavelet transform after reducing noise by minimum entropy deconvolution are compared with the results of EMD, EEMD, and VMD.

(5) Decomposition results of intermittent signals obtained by EMD

**Figure 14:**
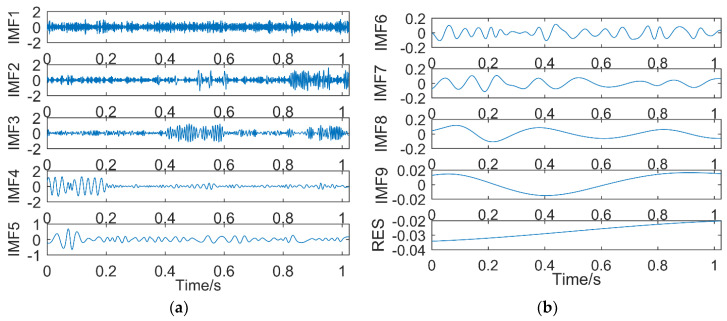
Decomposition results of intermittent signals obtained by EMD: (**a**) IMF1–IMF5; (**b**) IMF6–RES.

The intermittent signal is decomposed by EMD, and it is decomposed into nine sub-bands, including eight intrinsic mode functions and a residual. The decomposition results are shown in Figure 14. Figure 14a shows IMF1–IMF5, and Figure 14b shows IMF6–RES.

It can be seen from Figure 14a that IMF1 is the noise contained in the simulation signal, IMF2–IMF3 contains the frequency components of the simulation signals $y_{1}(t)$ and $y_{2}(t)$, and IMF4–IMF5 contains the frequency components of the simulation signals $y_{3}(t)$. However, the frequency mixing of IMF2–IMF5 is very serious. Figure 14b shows that the latter four IMFs and residual do not contain the frequency components. The decomposition result of EMD not only produces more invalid sub-bands, but also has serious frequency mixing of sub-bands with simulated signal frequency components. EMD is not suitable for the decomposition of intermittent signals.

(6) Decomposition results of intermittent signals obtained by EEMD

**Figure 15:**
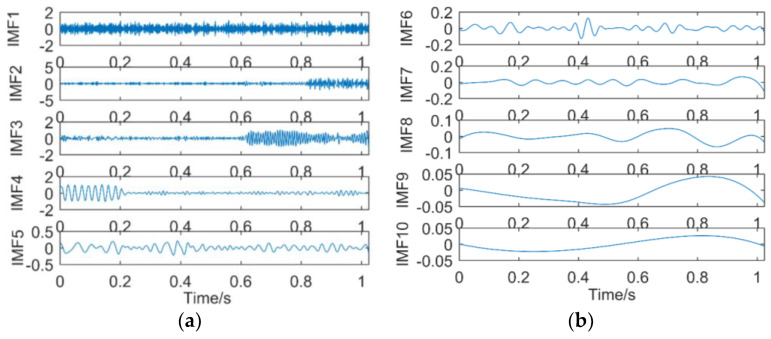
Decomposition results of intermittent signals obtained by EEMD: (**a**) IMF1–IMF6; (**b**) MF7–RES.

Figure 15 shows the decomposition result of the intermittent signals obtained by EEMD. Among them, the ratio of standard deviation of adding white noise to the standard deviation of the simulation signal is 0.2, and the number of adding white noise is 50 times. Figure 15a shows IMF1–IMF6, and Figure 15b shows IMF7–RES. The waveform of IMF1 is basically the same as the simulated signal. IMF2 is the Gaussian white noise. IMF3–IMF4 contains the frequency components of the simulation signals $y_{3}(t)$, but the mode mixing is serious. IMF5 contains the frequency components of the simulation signals $y_{1}(t)$. IMF6-–RES basically do not contain frequency components of the simulation signals. It is concluded that EEMD cannot decompose intermittent signals containing noise.

(7) Decomposition results of intermittent signals obtained by VMD

**Figure 16:**
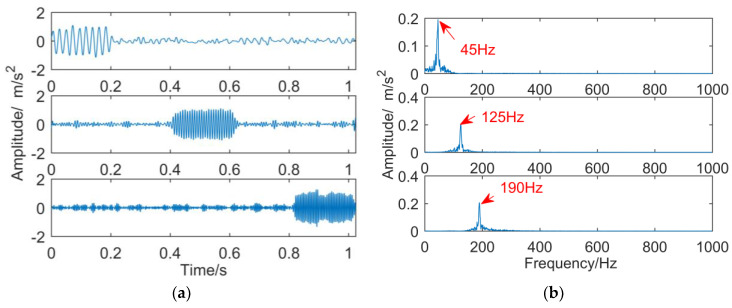
Decomposition results of intermittent signals obtained by VMD: (**a**) time domain; (**b**) frequency domain.

**Figure 17:**
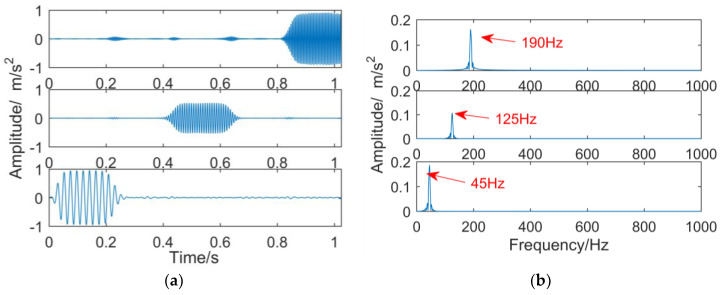
The sub-bands obtained by improved dual-tree complex wavelet transform after reducing noise by minimum entropy deconvolution: (**a**) time domain; (**b**) frequency domain.

When the number of decomposition layers is three and the penalty factor is 2000, the intermittent signal is decomposed by VMD, and the result is shown in Figure 16. From the time-domain waveforms of each sub-band in Figure 16a, three different time scales are decomposed in order from low to high frequency. However, apart from the characteristic components, each layer has a large amount of small fluctuations outside the intermittent harmonic vibration. The frequency components and the main frequency components are close to each other and can be classified as noise. The corresponding frequency domain in Figure 16b shows that the frequencies of 45, 125, and 190 Hz in the simulated signal are successfully extracted, but the center frequency of each sub-band is not smooth compared with Figure 17b.

(8) Decomposition results of intermittent signals obtained by IDTCWT

The simulation signal is decomposed by the improved dual-tree complex wavelet transform. The maximum decomposition level is eight and the adaptive decomposition level is four, so five sub-bands are obtained. By selecting the effective sub-bands adaptively and removing frequency mixing, the three sub-bands after reducing noise by minimum entropy deconvolution are obtained, as shown in Figure 17. Compared with the decomposition result of VMD, the frequency mixing of the improved dual-tree complex wavelet transform is improved greatly, and there are only some weak oscillations of the same frequency at the time domain waveform.

## 5. Experimental Verification

**Figure 18:**
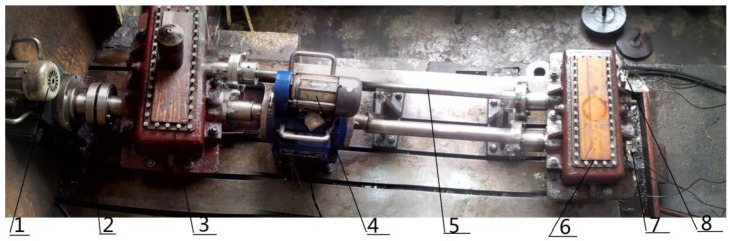
Experiment platform. 1—Speed-adjustable motor; 2—coupling; 3—accompanied gearbox; 4—speed reversing instrument; 5—torsion bar; 6—test gearbox; 7—acceleration sensor #1; 8—acceleration sensor #2.

**Figure 19:**
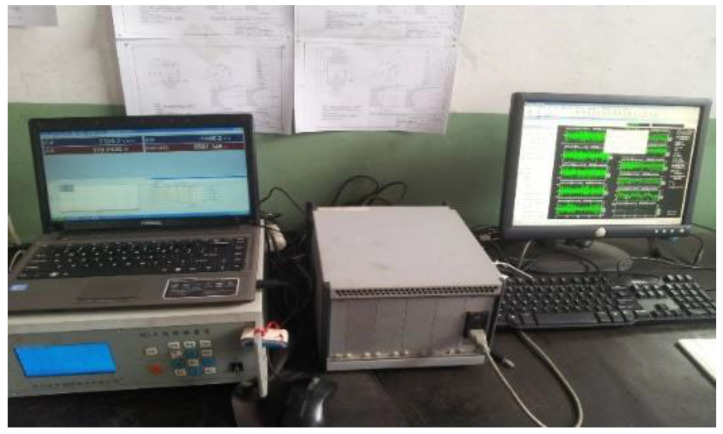
Console.

In order to verify the feasibility of improving the dual-tree complex wavelet transform in an engineering application, the closed power-flow gearbox test rig was used to carry out experiments. The composite vibration signals of the gearbox under normal conditions, bearing outer ring and ball fault conditions were measured by three-way acceleration sensors with model YD77SA, and good results were obtained. The closed power-flow gearbox test rig used in this paper is shown in Figure 18. The console is shown in Figure 19.

In the experiment, the gear box was loaded by the internal force generated by the torsion bar. The speed of the gearbox was adjusted by controlling the electromagnetic speed regulating the asynchronous motor, and the regulating range was 120–1200 r/min. The gear transmission test bench is shown in Figure 16. The experimental devices of the test bench mainly included test bearings, rotational speed displays, motors, test gears, rotating shafts, and three-way acceleration sensors. The experimental bearing model was 32212, and the three-way acceleration sensor model was YD77SA (sensitivity is 0.01). The faulty bearing was at the three-way acceleration sensor 1#. The fault frequency of the rolling element was 72 Hz, the fault frequency of the outer ring of bearing was 160 Hz, and the meshing frequency of the gear was 360 Hz. The specific parameters are shown in Table 3.

**Figure 20:**
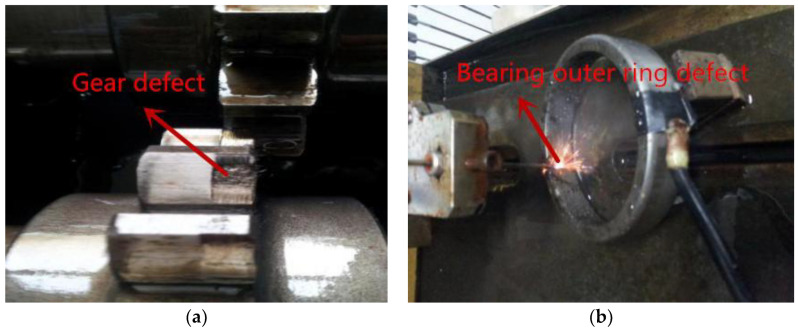
Bearing and gear fault diagram: (**a**) spalling failure of gear; (**b**) outer ring fault through EDM.

In this paper, the composite fault is taken as an example to verify the feasibility of improving the dual-tree complex wavelet transform. Fault types include gear pitting and rolling element failure, as shown in Figure 20.

### 5.1. Selection of Sensors

In order to obtain a more realistic vibration signal, the sensor used must have high precision and sensitivity, and have a better resistance to noise. After comprehensive consideration, the voltage output type piezoelectric accelerometer sensor with model YD77SA was selected. The sensor was composed of two parts, namely: a piezoelectric accelerometer and special ICP chip. The power supply and signal output shared a single cable (that is, the coaxial cable provided a constant current supply of 2–20 mA to the sensor, and the signal output was also through this cable). The constant current power supply could be directly connected to the recording and display instruments, which simplified the test system and improved the accuracy and reliability of the test. The sensor had the advantage of high precision, strong anti-interference ability, light weight, easy installation, and high cost performance, so it was selected. At present, the sensor has been applied on many occasions, such as fault diagnosis. The sensor parameters were as follows: sensitivity was 0.01 V/ms^2^, measurement range was 500 ms^2^, resolution was 0.002 ms^2^, and mass was 48 g.

### 5.2. Arrangement of Sensors Measuring Points

In order to reduce the attenuation of the signal during the transmission, the position of the sensor was closer to the vibration source when measuring the vibration signal. When the vibration source produced an impact, the vibration of the gear train was transmitted to the bearing through the shaft, and finally to the gearbox. Therefore, two accelerometers (#1 and #2) were added to the experiment. The data were collected from the accelerometer 1 #. The two sensors measured the vibration data of x, y, and z directions, respectively. All of the data used in this paper came from the sensors.

### 5.3. Experimental Signal Analysis

**Figure 21:**
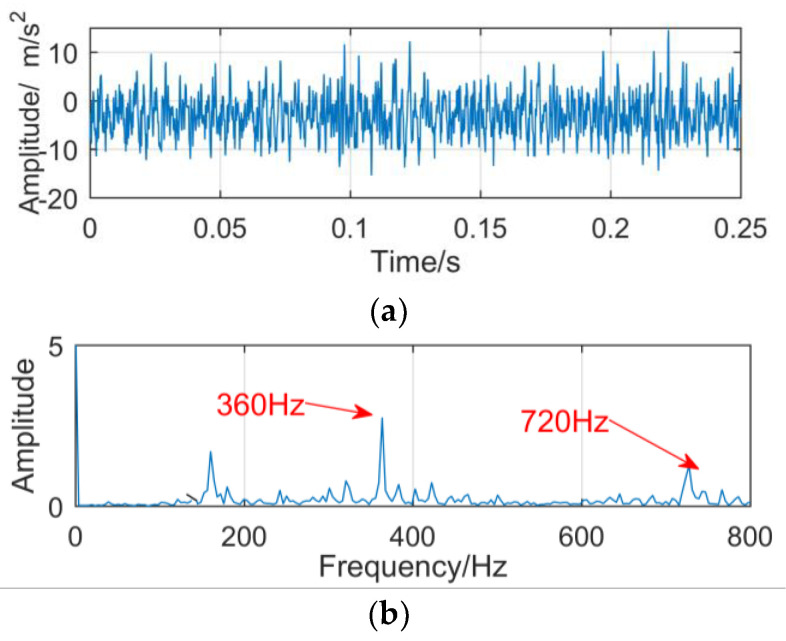
The vibration signal: (**a**) time domain; (**b**) frequency domain.

The vibration signal of the gearbox composite fault collected by the sensor is shown in Figure 21. Because of the influence of the strong background noise, only the meshing frequency (360 Hz) and its double frequency of gears appeared in the frequency spectrum, but the fault frequency (160 Hz) of the outer ring did not appear.

(1) Decomposition results of vibration signals obtained by EMD

**Figure 22:**
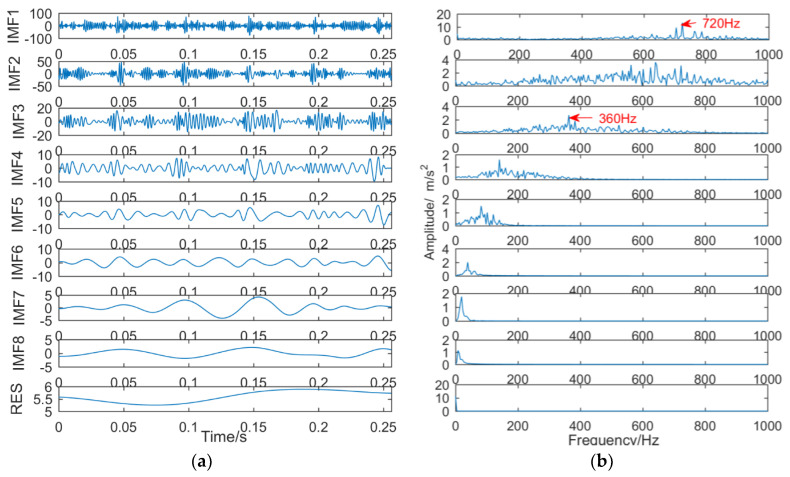
Decomposition results of vibration signals obtained by EMD; (**a**) time domain; (**b**) frequency domain.

The vibration signal was decomposed by EMD, and it was decomposed into eight IMFs and a residual. The result is shown in Figure 22. As illustrated in Figure 22a, IMF6–RES basically does not contain frequency components in the fault signals. From Figure 22b, IMF1 and IMF2 can identify the frequency of a gear spalling fault at 360 Hz and at its double frequency of 720 Hz. The frequency mixing of each IMF obtained by EMD is serious, and the bearing outer ring fault frequency of 160 Hz has not been successfully extracted.

(2) Decomposition results of vibration signals obtained by EEMD

**Figure 23:**
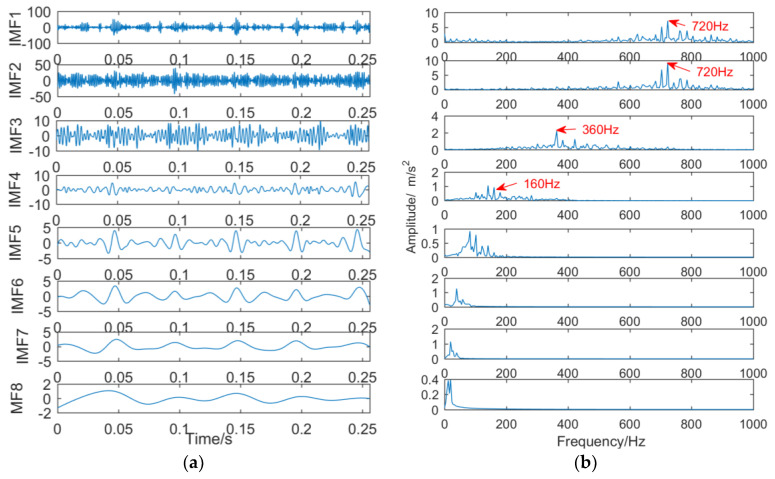
Decomposition results of vibration signals obtained by EEMD: (**a**) time domain; (**b**) frequency domain.

The vibration signal was decomposed into eleven IMFs and one RES by EEMD, and the first eight IMFs were selected. The result is shown in Figure 23. As can be seen from Figure 23a, there is less information in the IMF7–RES fault signal, and IMF1–IMF6 contains fault signal information, but the mixing is serious. From Figure 23b, the bearing outer ring fault frequency of 160 Hz and the gear peeling fault frequency of 360 Hz and its double frequency 720 Hz can be identified.

(3) Decomposition results of vibration signals obtained by VMD

**Figure 24:**
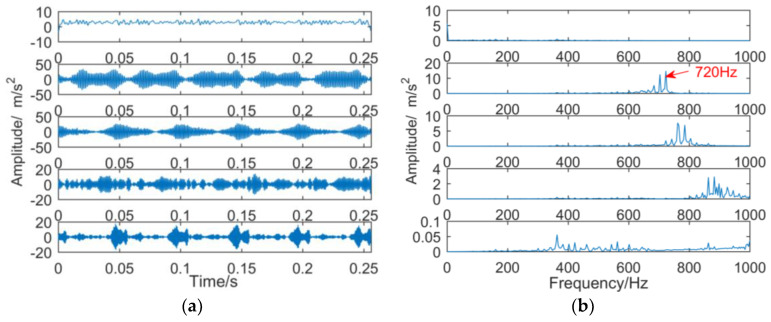
Decomposition results of vibration signals obtained by VMD: (**a**) time domain; (**b**) frequency domain.

When the number of decomposition layers is five and the penalty factor is 2000, the intermittent signal is decomposed by VMD, and the result is shown in Figure 24. The decomposition results show that only the double frequency of 720 Hz of the gear spalling fault appears in the second layer, and the outer ring fault frequency 160 Hz and the gear spalling fault frequency 360 Hz do not appear, indicating that the VMD decomposition effect is poor.

(4) Decomposition results of vibration signals obtained by IDTCWT

**Figure 25:**
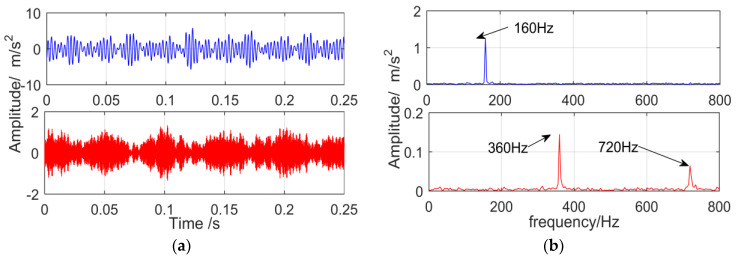
The sub-bands obtained by improved dual-tree complex wavelet transform after reducing noise by minimum entropy deconvolution: (**a**) time domain; (**b**) frequency domain.

The fault signal was decomposed by improved dual-tree complex wavelet transform. The sub-bands obtained by the improved dual-tree complex wavelet transform after reducing noise by minimum entropy deconvolution is shown in Figure 25.

By observing the frequency spectrum of each sub-band in Figure 25b, it is found that the improved dual-tree complex wavelet transform successfully extracted the fault frequency (160 Hz) of the outer ring, fault characteristic frequency (360 Hz), and double frequency of the gearbox.

## 6. Conclusions

In order to solve the limitation of the double-tree complex wavelet in fault diagnosis, such as the decomposition layer and mode mixing, this paper proposes an improved dual-tree complex wavelet, which is successfully applied to gearbox composite fault diagnosis. The improved dual-tree complex wavelet transform can adaptively determine the decomposition level and the effective sub-band. It can effectively extract the sub-band that has the greatest correlation with the original signal. The adaptive determination of the decomposition level and the adaptive selection process can minimize the influence of human factors. Furthermore, the improved dual-tree complex wavelet transform is used to remove the frequency mixing of each sub-band. The simulation results show that the proposed method can decompose not only the non-linear signals, but also the intermittent signals. The proposed method can extract the frequency of the intermittent signal perfectly at 190, 125, and 45 Hz, while EMD and EEMD cannot extract the frequency of the intermittent signal. The improved dual-tree complex wavelet transform combined with minimum entropy deconvolution is used to extract the fault features of the test signal, and the fault frequencies of the outer ring and outer ring of the gearbox are extracted successfully. The proposed method is applied to extract the gearbox faults, and the fault frequencies of gears at 360 Hz and bearing outer rings 160 Hz are successfully extracted. EMD and EEMD cannot extract the frequency of 160 Hz. The effectiveness of the proposed method is fully proven. It provides assistance for subsequent gearbox multi-fault feature extraction.

## Figures and Tables

**Table 1:** The relationship between the correlation coefficient size and correlation.

Correlation	Negative	Positive
Uncorrelated	−0.09 to 0	0 to 0.09
Weakly correlative	−0.3 to 0.1	0.1 to 0.3
Correlative	−0.5 to −0.3	0.3 to 0.5
Strongly correlative	−1.0 to −0.5	0.5 to 1.0

**Table 2:** The main frequency of each sub-band.

sub-band	1	2	3	4	5	6	7
frequency/Hz	700	430	100	100	45	20	20

**Table 3:** Experimental parameters.

Experimental Parameters	
Meshing method	Half tooth meshing
Transmission ratio	0 January 1900
Sampling frequency	8000 Hz
Sampling points	9 August 1905
Number of teeth	18 January 1900
Rotating speed	1200 rpm
Rotational frequency	20 Hz
Load torque	1000 N·m
Meshing frequency	360 Hz
Outer ring fault frequency	160 Hz
Ball fault frequency	72 Hz
